# Photosynthate accumulation in solar-powered sea slugs - starving slugs survive due to accumulated starch reserves

**DOI:** 10.1186/s12983-016-0186-5

**Published:** 2017-01-19

**Authors:** Elise M. J. Laetz, Victoria C. Moris, Leif Moritz, André N. Haubrich, Heike Wägele

**Affiliations:** 1 Zoological Research Museum Alexander Koenig, 162 Adenauerallee, Bonn, 53113 Germany; 2 Institute for Evolutionary Biology and Ecology, University of Bonn, An der Immenburg 1, Bonn, 53121 Germany

**Keywords:** Sacoglossa, Amylose, Elysia, Lugol’s Iodine, Kleptoplast, Starch

## Abstract

**Background:**

Solar-powered sea slugs are famed for their ability to survive starvation due to incorporated algal chloroplasts. It is well established that algal-derived carbon can be traced in numerous slug-derived compounds, showing that slugs utilize the photosynthates produced by incorporated plastids. Recently, a new hypothesis suggests that the photosynthates produced are not continuously made available to the slug. Instead, at least some of the plastid’s photosynthetic products are stored in the plastid itself and only later become available to the slug. The long-term plastid-retaining slug, *Elysia timida* and its sole food source, *Acetabularia acetabulum* were examined to determine whether or not starch, a combination of amylose and amylopectin and the main photosynthate produced by *A. acetabulum*, is produced by the stolen plastids and whether it accumulates within individual kleptoplasts, providing an energy larder, made available to the slug at a later time.

**Results:**

Histological sections of *Elysia timida* throughout a starvation period were stained with Lugol’s Iodine solution, a well-known stain for starch granules in plants. We present here for the first time, an increase in amylose concentration, within the slug’s digestive gland cells during a starvation period, followed by a sharp decrease. Chemically blocking photosynthesis in these tissues resulted in no observable starch, indicating that the starch in untreated animals is a product of photosynthetic activity.

**Conclusion:**

This suggests that kleptoplasts function as both, a nutritive producer and storage device, holding onto the polysaccharides they produce for a certain time until they are finally available and used by the starving slug to withstand extended starvation periods.

## Background

Sacoglossan sea slugs (Heterobranchia: Gastropoda) are also known as “solar-powered sea slugs” and “leaves that crawl” due to some members’ ability to steal chloroplasts (kleptoplasts) from their algal food and retain them for many months [1–5]. As the only described metazoans with this ability, they stand out as study subjects, captivating the interest of both researchers and laymen alike. Most sacoglossans feed on chlorophyte species by piercing the cell wall and sucking out the cell content including the chloroplasts [6–8]. While other organelles such as mitochondria are digested in all slug species, some slug species can incorporate and maintain chloroplasts in their digestive gland tubule cells. Because these stolen chloroplasts remain photosynthetically active, this incorporation phenomenon is called functional kleptoplasty [9].

Chlorophytes, the algae most often ingested by sacoglossans, produce numerous photosynthates including carbohydrates, lipids and proteins [3, 4, 10–13]. The main photosynthate produced however is starch, a combination of amylose and amylopectin [10]. To establish whether or not photosynthates are made available to a sacoglossan slug, radiolabeling studies with ^14^C or ^13^C were conducted [5, 14–19], finding that both photosynthetically and heterotrophically fixed carbon is present in multiple slug-derived compounds for some species. These include glucose, galactose and an unidentified third compound [20], pigments [18], mucus [17], amino acids, organic acids and others (not specified in [21]). Since the total amount of incorporated ^14^C was lower in specimens kept in the dark, which presumably has no effect on heterotrophically derived ^14^C, the amount of photosynthetically derived ^14^C was determined, demonstrating the metabolic movement of photosynthetically derived carbon into multiple slug-produced compounds ([15, 20–22], full review available in [9]). Recently two long-term plastid retaining (LtR) species, *Elysia timida* Risso 1818 and *Plakobranchus ocellatus* van Hasselt 1824, were shown to fix ^14^CO_2_ further showing that photosynthetically active plastids are fixing carbon, which is then incorporated into slug tissues [23]. Although CO_2_ fixation requires light, LtR species do not necessarily need light and can survive several months in complete darkness, although their longevity is not equal to that of specimens kept in the light. They also survive when photosynthesis is chemically blocked (e.g. monolinuron), although their longevity may be compromised [24].

These last observations question the necessity for kleptoplast functionality and the nature of the benefits gleaned by starving sacoglossans. Recent hypotheses consider sequestered chloroplasts as a source of stored food reserves rather than as a source of directly and continuously available photosynthates [24, 25].

Only one LtR form has been investigated to determine if photosynthates accumulate, the misfit species *Elysia chlorotica*, a slug that feeds on the heterokontophyte, *Vaucheria litorea* Agardh 1832 rather than chlorophyte species like most other sacoglossans. Pelletreau et al. [25] observed lipid droplet accumulation in *E. chlorotica’s* digestive gland tubules during a starvation period, and they state that these lipid droplets were likely “of algal origin”. Lipid droplets are known to be the main photosynthate storage molecule produced by *V. litorea*, so this accumulation is not surprising and lends evidence to the hypothesis regarding chloroplasts as photosynthate larders. It remains unknown whether chlorophyte-feeding sacoglossans profit from stored photosynthates in a similar manner or not. Starch grains have been seen in numerous electron micrographs [1, 5, 15, 26–28], however no information on starch quantity, production or accumulation has been reported.

In this paper, we examine the accumulation of starch within kleptoplasts in the digestive gland tubules of stenophagous, LtR *Elysia timida*. Starch, the main storage polysaccharide produced by the chlorophyte *Acetabularia acetabulum* Linneaus is a complex carbohydrate comprising glucose units bound together to form amylose and amylopectin [29–31]. This starch is stored in the form of granules within the chloroplast’s stroma. When needed by the algal cytosol, it is broken down to maltose and glucose units and transported to the algal cytosol, a process that occurs nightly when photosynthesis ceases [31]. Whether or not these processes function in kleptoplasts stored in the slug’s digestive gland remain uninvestigated. It is also still unclear whether any photosynthates produced are immediately transported to the slug or if the slug lacks the feedback system found in the algae, therefore directly causing a build up of starch within the kleptoplast. Any starch build up would support the more recent “larder hypothesis” [25, 32] - that photosynthesis does contribute to meeting a starving slug’s metabolic needs, but not through the continuous release of the photosynthates produced, but rather by storing them inside the plastid where they later become available to the slug when the plastids are finally digested at a later time point.

In order to test if the starch granules stained here were really produced by photosynthesis inside the slug and were not for instance thylakoid membrane degradation inside the tubules [33], we used the photosynthesis blocker, monolinuron (Algol), during the starvation process. Phenylureas such as monolinuron bind at the site of the quinone B in the D1 protein of photosystem II (P680) and thus block the electron flow [34]. Therefore, by adding this inhibitor from the beginning of the starvation process, we hypothesized that we would not observe any starch increase if starch is indeed a product of ongoing photosynthesis in the slug. Pulse Amplitude Modulated (PAM) fluorometry values were also measured in both, monolinuron treated and in untreated samples, to monitor photosynthetic efficiency during the starvation process [7, 32, 35]. Since *Acetabularia acetabulum* availability in the Mediterranean is highly variable [36], both a spring and fall population were investigated to account for seasonal differences due to temperature, food availability or other factors.

## Methods

*Elysia timida* and *Acetabularia acetabulum* were collected in Blanes, Spain (November 2014) and Fetovaia, Elba, Italy (April 2015) at one to two meters depth. Specimens were kept in groups of 25, in aerated tanks containing roughly 3 L freshly prepared artificial seawater (changed every other day). Artificial lighting for 12 h each day provided full-range lighting measuring about 220 μE^−2^s^−1^ at the water’s surface to mimic natural conditions and the optimal irradiance [36, 37]. Both populations were kept in the laboratory at different temperatures, 20–22 °C average for the spring population, and 18 °C for the fall to match the water temperature at collection and mimic natural conditions. All specimens were kept in the tanks with *A. acetabulum* for two weeks to acclimate to laboratory conditions and to assure feeding.

Prior to fixation, the maximum quantum yield (PAM or Fv/Fm) was measured three times with a Pulse Amplitude Modulated fluorometer (Diving PAM) for each animal in order to compute a mean value (for detailed methods, see 35). This method assesses photosynthetic efficiency in living tissues. PAM values were also measured for specimens treated with the photosynthetic inhibitor Monolinuron – diluted to 2 μg.ml-1 with filtered seawater, in order to determine if photosynthesis was still occurring in the slugs and how it decreased with the continuous presence of the blocker. The monolinuron and seawater mixture was prepared freshly and changed with the water every other day to ensure continuous exposure.

**Fig. 1:**
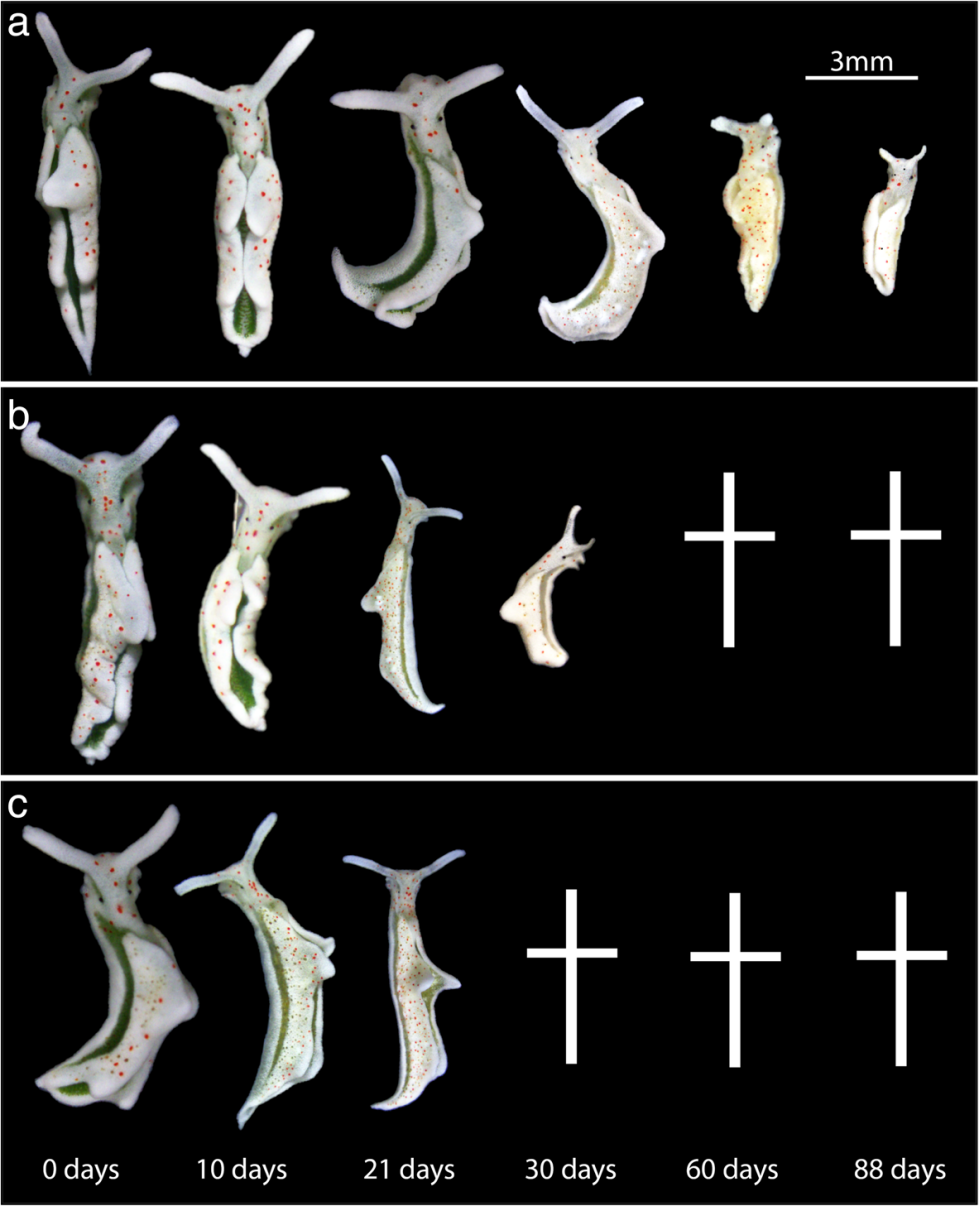
Starvation in *E. timida* Populations. **a** Series of photos showing starving *E. timida* from Blanes, Spain. **b** Starvation photos of *E. timida* from Elba, Italy. **c** Starvation in monolinuron-treated specimens. A cross (†) indicates that animals from this population did not survive starvation up to the day indicated

**Fig. 2:**
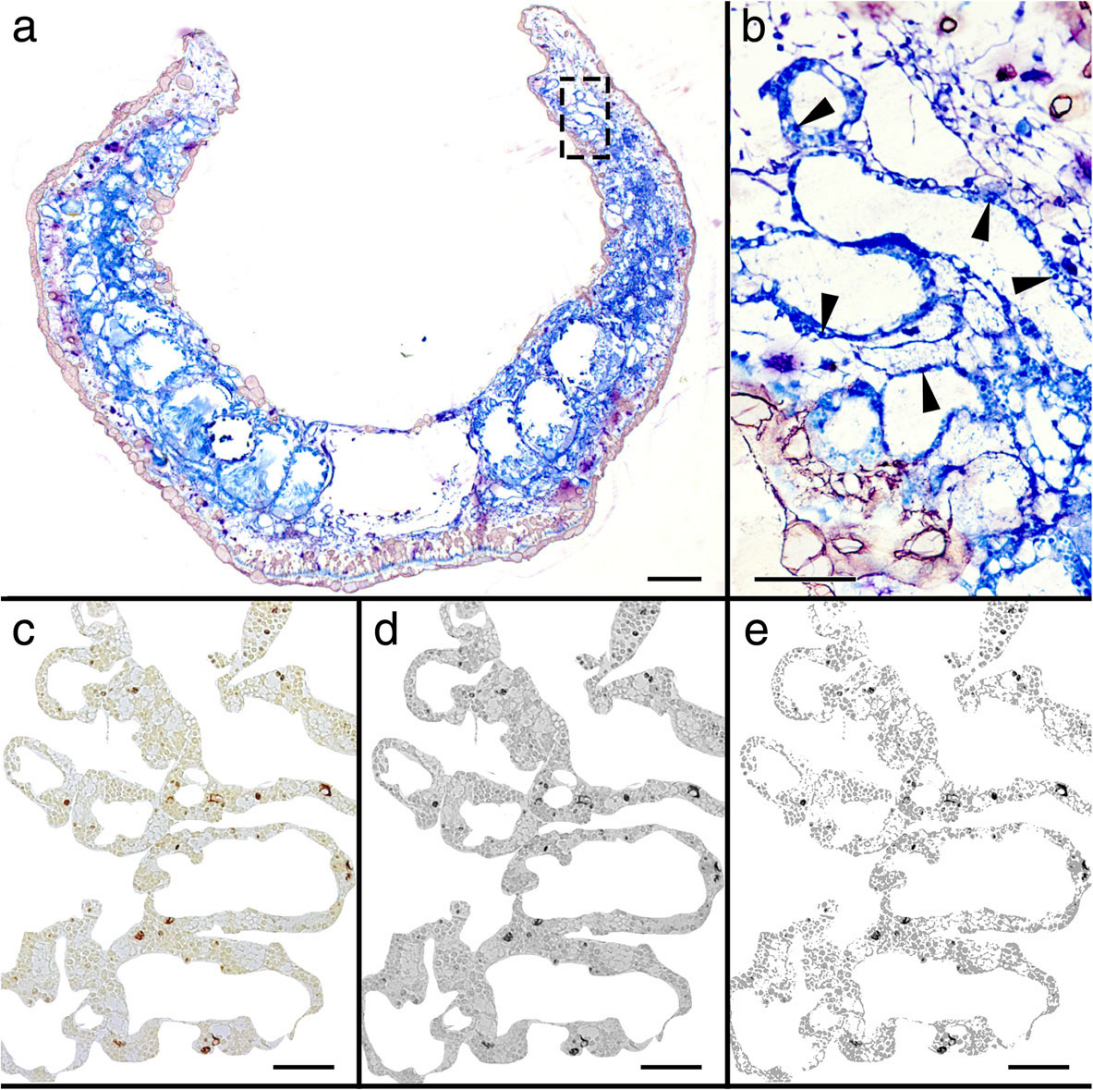
Methods. **a** Toluidine-blue stained cross-section of 42 days starvation specimen of *E. timida* from Blanes population, the rectangle indicates the area seen in B; (**b**) magnified area showing digestive tubule epithelium with chloroplasts (tubules indicated with arrowheads) in the parapodia at higher magnification; (**c**) Lugol’s solution-stained section of a 0 days starved *E. timida* from the Elba population, the digestive gland tubules have been isolated by deleting the background, non-digestive tissue; (**d**) Conversion to greyscale image in Adobe Photoshop; (**e**) *Grey* shades reduced to 5 for final starch counting, the *dark black* coloration shows the Lugol’s stained starch. Scale bars: A = 50 μm; B = 25 μm; C-E = 20 μm

**Fig. 3:**
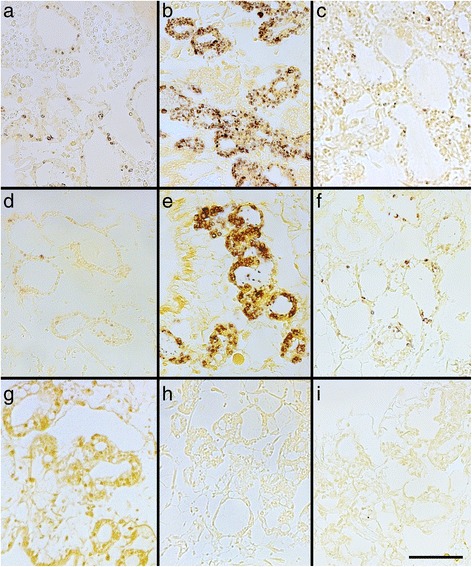
Starch Accumulation and Degradation in Various *E. timida* Specimens.**a**-**c** *E. timida,* fall population – Blanes, (**a**) starved for 0 days; (**b**) starved 42 days; (**c**) starved 88 days; (**d**-**f**) *E. timida,* spring population – Elba, (**d**) starved 0 days; (**e**) starved 10 days; (**f**) starved 30 days; (**g**-**i**) monolinuron-treated *E. timida,* spring population – Elba, (**g**) starved 3 days; (**h**) starved 10 days; (**i**) starved 21 days. Scale bar 50 μm

Forty individuals were starved for each time series: the fall population, spring population and monolinuron. Two specimens (fall population) or three specimens (spring population) were first photographed (Fig. 1) and then fixed in 4% formaldehyde for the following starvation time points given in days: 0, 3, 10, 15, 21, 30, 42, 88. The 0-day individuals were preserved as a control group, to measure the amount of starch in the incorporated chloroplast when the slug is normal, unstarved conditions. These animals were removed directly from the algae for fixation. Each specimen was fixed at 17:00 to maximize the amount of starch produced before it is broken down at night [31]. After fixation, specimens were embedded in hydroxymethacrylate (Kulzer Technovit® 7100) and sectioned at 2.5 μm thick. Every third section was stained with toluidine-blue in order to observe the overall anatomy of each specimen and assure it was not a juvenile (Fig. 2a, b) while the next sections were stained with a 1:10 Lugol’s Iodine Solution (Fisher Scientific) diluted in distilled water in order to quantify amylose (Figs. 2c and 3a-i). Lugol’s solution has long been used by botanists to identify starch in plants and algae, with Werz and Clauss [30] successfully identifying it in *A. acetabulum.*

Ten regions were chosen throughout the slug, in order to analyze different areas containing digestive gland tubules. For each region, two pictures were taken and analyzed in order to estimate the mean relative starch percentage value (RSP). This percentage value was computed using Adobe Photoshop (version CS5.1) by first deleting all non-digestive gland tubule tissue and recording the total digestive gland tubule area (Fig. 2c). Then each image was converted to greyscale (Fig. 2d), reduced to 4–5 shades depending on the staining intensity (Fig. 2e), and the two darkest colors – those corresponding to the amylose stained tissue – were measured in pixels. Each image was adjusted individually to avoid overestimating the relative starch percentage (RSP). These measurements were then converted back to area measurements from pixels based on the original image scale.

## Results

**Fig. 4:**
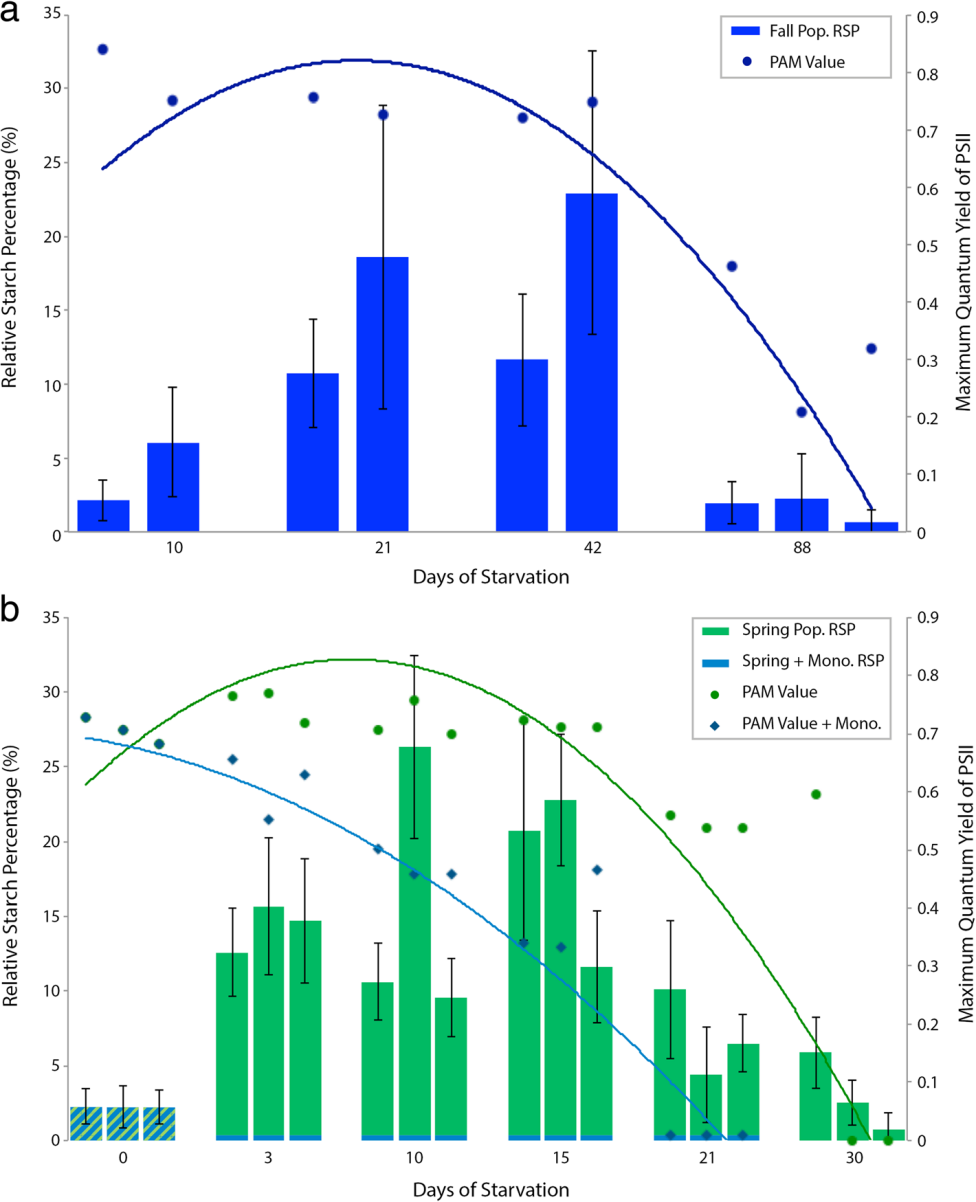
Histogram of the Mean Relative Starch Percentage Values (MRSP) ± Standard Error. **a** *E. timida* specimens of the Spain fall population (MRSP on the first y axis, left side) at different starvation time points, 10 days, 21 days, 42 days and 88 days and their relative maximum quantum yield of PSII with a parabolic trend line (on the second y axis, right side). **b** MRSP ± standard error for each *E. timida* specimens of the Elba spring population represented as the histogram (MRSP on the first y axis, left side) at different starvation time points, 0 day, 3 days, 10 days, 21 days, and 30 days and their relative maximum quantum yield of PSII with a parabolic trend line represented by the line (on the second y axis, right side). Specimens under starvation conditions (S) are represented in green, while those in starvation and monolinuron treatment (S + M) at the same time points are represented in blue for both RSP and maximum quantum yield of PSII. Specimens for 0 day of starvation are the same S and for S + M and therefore are hatched with *blue* and *green*. The specimens treated with monolinuron under starvation have a zero relative starch percentage value

**Fig. 5:**
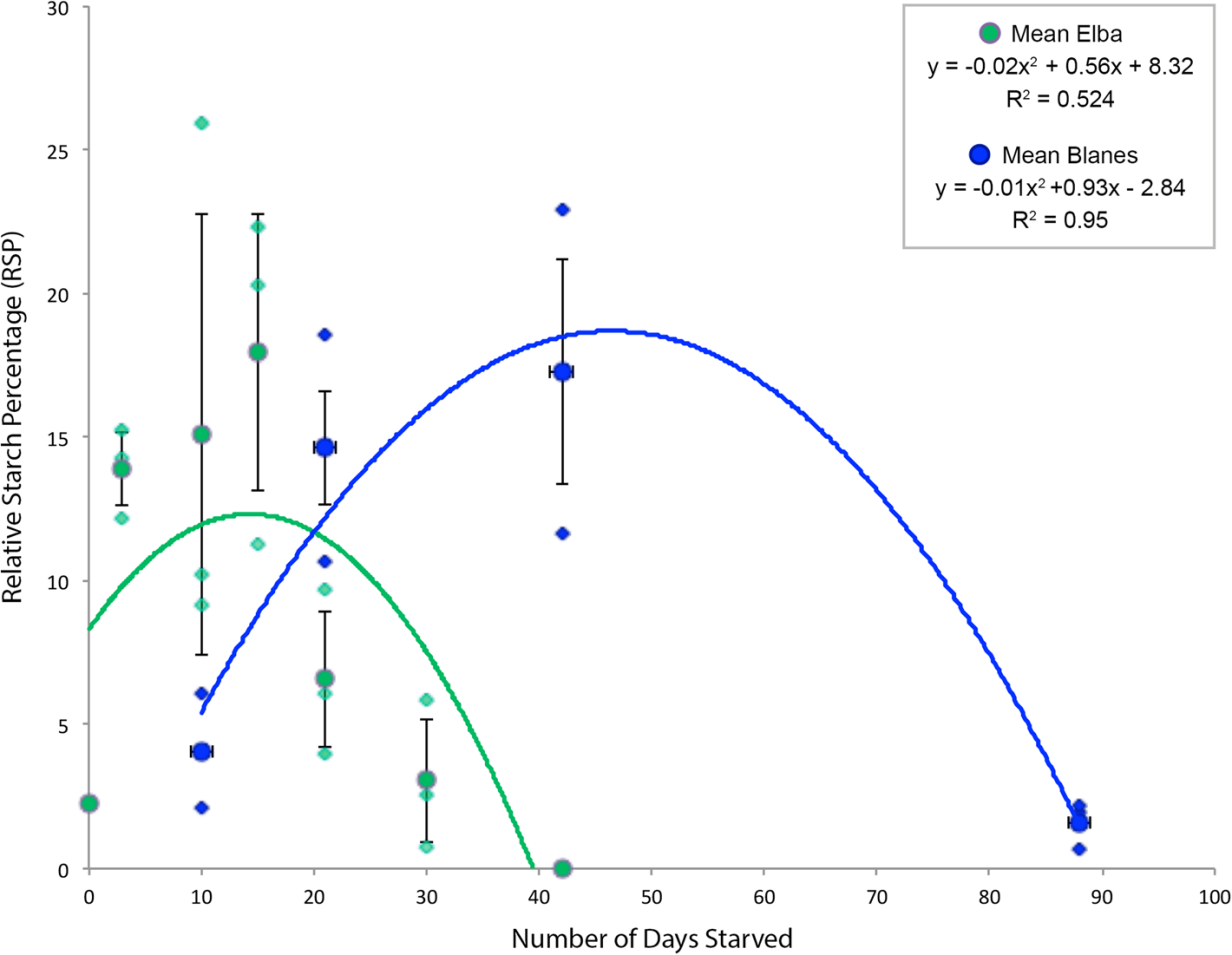
Overall Relative Starch Percentage in both Populations. The MRSP for the Blanes, fall population slugs is shown in blue and the Elba, spring population is shown in green (including two specimens which starved for 42 days). Best fit curves show the shift in total days starved between the two populations, and show a higher overall RSP in the Blanes fall population

Both populations surveyed here show first an increase in amylose followed by a decrease compared to the 0-day, unstarved control specimens (Figs. 3 and 4). Only the specimens from Blanes (Spain, collected in November) survived 88 days of starvation. The *E. timida* specimens from Elba (Italy, collected in April) did not survive more than 30 days of starvation (except for some specimens that managed 42 days) (Figs. 4 and 5).

In the case of the Blanes fall population, the relative starch percentage (RSP) increases until 42 days (Fig. 3a,b), reaching a maximal 23% tubule area coverage. This value drops to a mean of 2.2% after 88 days of starvation (Fig. 3c). In the same specimens, the maximum quantum yield of PSII values, which averaged 0.75 at the beginning, decreased to an average 0.30 (Fig. 4a).

The Elba spring population also showed an increase in the RSP until 15 days of starvation (Fig. 3d-f). Although the maximal value, 25.91 is reached after 10 days of starvation in one specimen, the mean value is at 15 days of starvation (17.95), and is higher than the average for 10 days (15.1). After 21 days of starvation, the RSP decreases and reaches a mean of 2.5 after 30 days. The maximum quantum yield of PSII (PAM) follows this trend. These PAM values are stable at the beginning of the starvation period and oscillate around 0.70 up to 21 days of starvation when they decrease to a mean of 0.55 and finally drop to values close to 0 (in two specimens) (Fig. 4b).

Monolinuron-treated samples did not contain amylose, at any time point during the starvation period (starting after the 0-day, control time point) (Figs. 3g-i and 4b). Moreover, the maximum quantum yield of PSII (PAM) values decreased faster during starvation in specimens treated with monolinuron, than in those without. After 10 days the mean value was 0.47 and after 21 days most of them had a zero value and began to die (Fig. 4b).

## Discussion

This study clearly reveals starch granules in starving *E. timida* adults, showing that this photosynthate is produced and maintained by *A. acetabulum* kleptoplasts. Unstarved (control) slugs contained only small amounts of starch. After 3 days (in the case of the Elba spring population) or 10 to 21 days (Blanes fall population), incorporated chloroplasts had the time to produce photosynthates, which accumulated as starch within the plastid (Fig. 5). After 21 days (Elba population) and 42–88 days (Blanes population), the RSP decreased, indicating that more starch is degraded than produced. Since the PAM values decrease at the same times as the RSP for both populations, a decrease in the electron chain transfer and therefore in the photosynthetic activity can be inferred. Moreover, the small amount of starch remaining after extended starvation indicates that this molecule is likely degraded and consumed by either the slug or the plastid itself, to meet its own metabolic needs. A quantitative number of chloroplasts at each time point is unavailable in published literature, although Laetz et al. [38] show a decrease in the number of functional plastids, based on chlorophyll a autofluorescence, throughout a starvation period. While they could not interpret the decrease in functional chloroplasts as a decrease in plastids themselves, since the plastids could still be there without intact chlorophyll a, the combination of this information and the decrease in starch presented here suggests the chloroplasts are no longer there and therefore likely being digested [38].

Even though the two populations analyzed show the same trend, an increase in starch followed by a decrease, the RSP maxima and minima do not occur at the same time point (Fig. 5). The fall population produced starch slowly and was able to survive longer starvation periods, whereas the spring population produced a high amount of starch very quickly at the beginning of the starvation process and died sooner. This was likely caused by temperature differences due to the time of year. The population from Blanes was starving during the fall with a lower temperature in the laboratory (18 °C) than the population from Elba (20–22 °C). Therefore, the chloroplasts in specimens from Elba probably demonstrated a higher metabolic rate, producing starch faster. This higher activity could also explain the shorter lifespan of the starved specimens from Elba compared to those from Blanes, since the chloroplasts may be degraded more quickly making them less functional overall.

Another effect due to the sampling period is that *A. acetabulum* was not found in the same quantity and not in the same point in its life cycle. The fall population (Blanes) was found amongst *A. acetabulum* that were young and completely uncalcified whereas the spring population (Elba) was collected from algae that had already grown long stalks and were already forming caps [39]. This almost aligns with the results from Marin and Ros [36], who described cap-formation and lower stalk calcification in Mazarrón Bay, Spain during February. We observed cap formation on partially calcified *A. acetabulum* during April, on the island of Elba, Italy. Calcified *A. acetabulum* stalks present a physical barrier that prevents *E. timida* from feeding [39]. Cap-formation is the last step in the *Acetabularia* lifecycle and is followed by a planktonic stage that presumably cannot be eaten by *E. timida.* Calcified stalks and cap-formation indicate a lower amount of food meaning slugs take in fewer kleptoplasts within the spring population (Elba) slugs, which may also account for a faster decrease in the spring RSP. Kleptoplast abundance has not been described from adult specimens collected in different seasons, so this hypothesis warrants further investigation.

The immediate decrease in amylose observed in monolinuron-treated specimens shows that starch production is the result of the ongoing photosynthesis in incorporated chloroplasts. Furthermore, the slugs are not able to face longer starvation periods and show drastically reduced longevity, dying after 21 days. This contradicts previous findings [23, 24],which suggested that monolinuron-treated animals and those kept in the dark lost weight and showed survival rates almost indistinguishable from those starving in illuminated conditions. However, the specimens used in Christa et al. [24] were bred and raised in a climate-controlled system rather than wild-caught animals kept in a temperature fluctuating environment reflecting the natural habitat, and this or other unknown factors may have influenced our results. Monolinuron may also have unknown side effects, which might compromise the longevity of marine animals, however no peer-reviewed investigations into this issue were found and the manufacturer of this compound states it is safe for animals. The validity of this statement should be verified in future studies involving organisms that do not depend on photosynthesis for their nutrition.

Christa et al. [32] state that monolinuron-treated slugs have a “marginally reduced” life expectancy where chemical blocking is likely incomplete. They continue by saying that “some plastids may still be able to fix carbon to a small extent, contributing energy-rich polymers that are available to the slugs”, but this was not seen to be the case in this report, for *A. acetabulum* plastids and starch production in *E. timida.* The exact effects of monolinuron in these tissues is still unknown, and it is possible that these plastids may still be producing simple sugars such as fructose and glucose, the prerequisite components of amylose and amylopectin, but not producing starch itself. If starch was produced in some of the unblocked plastids, as Christa et al. [32] suggest, it should have been detected in the samples presented here. We therefore conclude that starch is not produced in monolinuron-treated slugs. The decreased longevity seen in our samples treated with monolinuron leads us to conclude that starch produced in these kleptoplasts does help prolong a starving slug’s life. Yet, the specific effects of monolinuron on all photosynthetic production will require further research.

By demonstrating that *E. timida* specimens were able to incorporate photosynthetically active chloroplasts, which increase their lifespan during starvation via starch production, we conclude that these incorporated chloroplasts provide nutrition and appear essential for slugs in longer starvation periods. The slug as a direct recipient of these photosynthates is a more likely scenario, since the isotopic radio labeling studies showed ^14^C labeled carbon in numerous slug produced compounds [5, 14–18], however, it remains difficult to know exactly when the slugs have access to this additional source of energy during starvation. It is likely that the slug facing starvation will have access to these photosynthates when the kleptoplasts start to degrade on their own, or are actively digested by the slug itself, at a certain time during starvation. Regarding our results, it seems likely that this degradation process of chloroplasts and their photosynthates starts at leasts when we observed the decrease in amylose and probably already beforehand. In order to fully understand this degradation process, the expression of the genes involved in digestion of chloroplasts and photosynthates during starvation should be investigated.

## Conclusions

This study is the first to examine starch production by the chloroplasts stolen and stored in long-term retaining sacoglossan slugs. Previous findings have uncovered algal-derived carbon in slug tissues unrelated to digestion, however it was unknown when and in which form fixed carbon was made available to the starving slug. We now know that not all of the photosynthates produced are immediately transported to slug tissue, rather some accumulate in the kleptoplasts as starch grains. These starch grains increase in size until about halfway through the maximum starvation time experienced by the slugs and subsequently decrease rapidly. This suggests that starving slugs receive the nutritive benefits of the enslaved plastids after an extended time period and that these chloroplasts do indeed help the slug withstand long starvation periods. By using the photosynthetic blocker monolinuron and finding the chloroplasts lack starch, we confirm here that the starch buildup is in fact due to photosynthesis occurring in these chloroplasts. While many aspects of this enigmatic interaction between slugs and kleptoplasts remain unknown, this study directly indicates a major benefit the slug receives from its sequestered chloroplasts. This directly supports the causative hypothesis that some species of sacoglossan slugs can withstand extended starvation due to their sequestered plastids.
